# Selenoprotein MsrB1 promotes anti-inflammatory cytokine gene expression in macrophages and controls immune response *in vivo*

**DOI:** 10.1038/s41598-017-05230-2

**Published:** 2017-07-11

**Authors:** Byung Cheon Lee, Sang-Goo Lee, Min-Kyung Choo, Ji Hyung Kim, Hae Min Lee, Sorah Kim, Dmitri E. Fomenko, Hwa-Young Kim, Jin Mo Park, Vadim N. Gladyshev

**Affiliations:** 1 College of Life Sciences and Biotechnology, Korea University, Seoul, 02841 South Korea; 2 Division of Genetics, Department of Medicine, Brigham & Women’s Hospital and Harvard Medical School, Boston, MA 02115 USA; 3 Cutaneous Biology Research Center, Massachusetts General Hospital and Harvard Medical School, Charlestown, MA 02129 USA; 4 Program in Cellular and Molecular Medicine, Boston Children’s Hospital, Department of Microbiology and Immunobiology, Harvard Medical School, Boston, MA 02115 USA; 5 Department of Biochemistry and Redox Biology Center, University of Nebraska, Lincoln, NE 68588 USA; 6 Department of Biochemistry and Molecular Biology, Yeungnam University College of Medicine, Daegu, 42415 South Korea

## Abstract

Post-translational redox modification of methionine residues often triggers a change in protein function. Emerging evidence points to this reversible protein modification being an important regulatory mechanism under various physiological conditions. Reduction of oxidized methionine residues is catalyzed by methionine sulfoxide reductases (Msrs). Here, we show that one of these enzymes, a selenium-containing MsrB1, is highly expressed in immune-activated macrophages and contributes to shaping cellular and organismal immune responses. In particular, lipopolysaccharide (LPS) induces expression of MsrB1, but not other Msrs. Genetic ablation of MsrB1 did not preclude LPS-induced intracellular signaling in macrophages, but resulted in attenuated induction of anti-inflammatory cytokines, such as interleukin (IL)-10 and the IL-1 receptor antagonist. This anomaly was associated with excessive pro-inflammatory cytokine production as well as an increase in acute tissue inflammation in mice. Together, our findings suggest that MsrB1 controls immune responses by promoting anti-inflammatory cytokine expression in macrophages. MsrB1-dependent reduction of oxidized methionine in proteins may be a heretofore unrecognized regulatory event underlying immunity and inflammatory disease, and a novel target for clinical applications.

## Introduction

Methionine sulfoxide reductase (Msr) plays a critical role in redox regulation of proteins; it reduces methionine sulfoxide residue, a product of methionine oxidation, thus converting it back to methionine. This contrasts with the action of many other antioxidant enzymes, which act on oxidized cysteine residues^1–5^. Methionine sulfoxide occurs as two diastereomeric forms, methionine-*S*-sulfoxide (Met-*S*-SO) and methionine-*R*-sulfoxide (Met-*R*-SO). These distinct substrates are stereospecifically reduced by MsrA and MsrB, respectively^6–8^.

Mammals possess one MsrA and three MsrBs (MsrB1, MsrB2, and MsrB3) genes. These enzymes are localized in different subcellular compartments: MsrB1 in the cytosol and the nucleus; MsrB2 in the mitochondria; MsrB3 in both the mitochondria and the endoplasmic reticulum; and MsrA in the cytosol, the nucleus, and the mitochondria^9^. MsrB1 is a selenoprotein that contains selenocysteine in place of the catalytic cysteine and thus exhibits a high catalytic efficiency. MsrB1 is mainly known for its antioxidant and protein repair functions^10^. However, emerging evidence supports its role as a switch for protein function via reversible oxidation/reduction of specific methionine residues^11–13^. In particular, MsrB1 was reported to regulate actin assembly in conjunction with Mical protein. Mical oxidizes two conserved methionine residues of actin to Met-*R*-SO, resulting in disassembly of actin polymer, whereas MsrB1 reduces Met-*R*-SO back to methionine and thus facilitates actin polymerization^13^. It was shown that actin cytoskeleton dynamics was defective in MsrB1-deficient bone marrow-derived macrophages (BMDMs)^13^, although it remains unclear which biological processes require MsrB1- and Mical-mediated regulation of actin assembly in macrophages.

Macrophages serve a variety of physiological functions, such as orchestrating innate and adaptive immune responses, clearing cell debris, and coordinating tissue development and homeostasis^14, 15^. While engaged in these processes, macrophages produce a multitude of cytokines in response to stimulation by molecules associated with tissue damage and microbial infection. Some of these cytokines promote inflammation and activate specific arms of immune effector mechanisms. Other cytokines exert anti-inflammatory effects, limiting the magnitude and duration of the inflammatory response. In a properly functioning immune system, the production of pro- and anti-inflammatory cytokines is subject to tight control; its balance is crucial for ensuring optimal immune defense and tissue maintenance as well as avoiding excessive and self-destructive inflammatory reactions. It remains incompletely understood how macrophages interpret their physiological state and external stimuli from the environment, and integrate this complex information into biochemical mechanisms governing cytokine gene expression^14^.

Numerous aspects of macrophage function are mechanistically related to actin cytoskeleton dynamics and redox metabolism^16, 17^. Macrophage responses to microbial pathogens and damaged tissue involve, for example, phagocytosis, intracellular vesicle trafficking, cell migration, and the production of reactive oxygen and nitrogen species. All of these molecular events have been demonstrated to influence cytokine production by macrophages^18, 19^. Given the role of Msr in both actin polymerization and actin Met-*R*-SO reduction, it is highly likely that Msrs contribute to shaping macrophage immune responses by modulating cytokine production. In this study, we investigated lipopolysaccharide (LPS)-induced cytokine expression in macrophages lacking Msr proteins. We also examined the effects of Msr deficiency on inflammatory responses in mice. Our findings revealed a role for MsrB1 in anti-inflammatory cytokine gene expression in macrophages and the control of acute inflammation *in vivo*.

## Materials and Methods

### Animals

MsrB1 knockout (KO) and MsrA KO mice were previously described^20, 21^. They were backcrossed with C57BL/6 mice (The Jackson Laboratory) for seven generations and then used for further studies as described below. MyD88 KO mice were from the *Myd88* ^*tm1.1Defr*^ line (The Jackson Laboratory). All procedures were approved by the University of Nebraska-Lincoln and Brigham and Women’s Hospital Institutional Animal Care and Use Committees and conformed to the NIH Guide for the Care and Use of Laboratory Animals.

### Reagents

LPS and 12-*O*-tetradecanoylphorbol-13 acetate (TPA) were purchased from Sigma-Aldrich. Mouse recombinant interleukin (IL)-1 and CD40L were purchased from R&D Systems. Pam_3_CSK_4_ and dextran sulfate sodium (DSS) were purchased from InvivoGen and MP Biomedicals, respectively.

### Primary cell culture and treatment

Primary macrophages were prepared by culturing C57BL/6 mouse bone marrow cells in Dulbecco’s modified Eagle’s medium (DMEM) with high glucose (Thermo Fisher Scientific) supplemented with FBS (10%), L-glutamine (2 mM), sodium pyruvate (1 mM), penicillin (50 U/ml), streptomycin (50 μg/ml), and recombinant mouse macrophage-colony stimulating factor (10 ng/ml; Peprotech) for 7 days. BMDMs were treated with LPS (100 ng/ml). Primary keratinocytes were isolated as described^22^ and were exposed to UVB (75 mJ/cm^2^). Primary dendritic cells were prepared as described^23^ and were treated with Pam_3_CSK_4_ (1 μg/mL) or CD40L (1 μg/mL). Intestinal epithelial cells were isolated from mice orally administered DSS as previously described^24^. Fibroblasts were cultured in DMEM (Thermo Fisher Scientific) and were treated with IL-1 (20 ng/ml).

### RNA analysis

Total RNA was extracted with Trizol (Thermo Fisher Scientific). RNA analysis by quantitative PCR was performed as described^25^ using the primers listed in Supplementary Table S1.

### Western blot analysis

Whole cell lysates were prepared and analyzed as described^25^. Immunoblot analysis was performed using antibodies to the following proteins: p-ERK (9101), p-JNK (9251), p-p38 (9211), p-S6 (4858; all from Cell Signaling Technology); STAT1 (sc-346), p-STAT1 (sc-8394), STAT3 (sc-482), p-STAT3 (sc-8059), IκBα (sc-371; all from Santa Cruz Biotechnology); and actin (A4700; Sigma-Aldrich).

### Cytokine analysis

Eight-week-old female mice were intraperitoneally injected with LPS (10 µg/g). Four hours after LPS treatment, mouse sera were collected, and then the plasma samples were sent for cytokine analysis using ELISA (AssayGate, Inc.). Plasma IL-1α, IL-1β, IL-1RA, IL-6, IL-10, IL-12p70, GM-CSF, and TNF-α were analyzed from independent mice.

### Acute inflammation model

One μg of 12-*O*-tetradecanoylphorbol-13-acetate (TPA) in 20 μl acetone was applied to the left auricle and 20 μl of acetone to the right auricle of mice. Ear thickness was measured 24 hr after treatment and ear tissues were preserved in 4% paraformaldehyde. Fixed skin samples were embedded in paraffin, sectioned at 5 μm, were stained with hematoxylin and eosin (H&E).

### Databases

Gene expression profiles of *MSRA*, *MSRB1*, *MSRB2*, and *MSRB3* in various cell lines, tissues, and conditions were examined by using the BioGPS database (Dataset: GeneAtlas MOE430, gcrma; Probesets: 1448856_a_at (*MSRA*), 1418888_a_at (*MSRB1*), 1424433_at (*MSRB2*), 1439151_at (*MSRB3*))^26^. Adult (10- to 12-week-old) mouse tissue samples were independently generated from two groups of four male and three female *C57BL*/*6* mice by dissection, and used for microarray analysis. Those information of all gene expression profile is available in the BioGPS database website (http://biogps.org).

### Statistical analysis

Data values are expressed as mean ± standard deviation. P values were obtained with the unpaired, two-tailed Student t-test.

## Results

### High expression of *MSRB1* in macrophages stimulated with LPS

**Figure 1:**
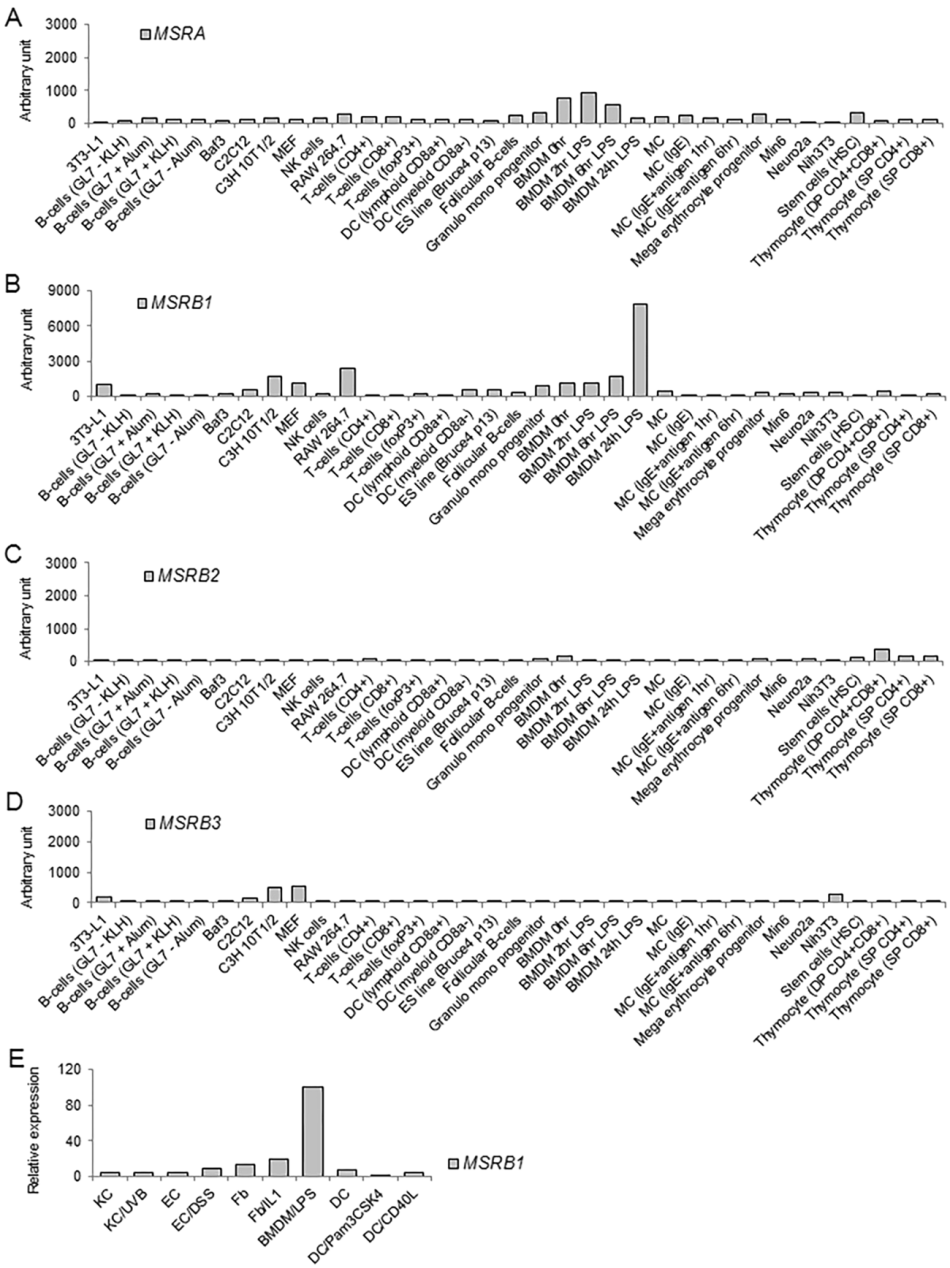
MsrA, MsrB1, MsrB2, and MsrB3 mRNA expression analysis in various mouse primary cell types and cell lines. (**A**–**D**) Comparison of relative amounts of mRNA encoding mouse MsrB1 (**A**), MsrA (**B**), MsrB2 (**C**), and MsrB3 (**D**) in the cell types and cell lines annotated in the BioGPS database (http://biogps.org/#goto = welcome). (**E**) Relative amounts of mouse MsrB1 mRNA in keratinocytes (KC) with or without ultraviolet-B irradiation (UVB; 75 mJ/cm^2^), enpithelial cells (EC) from intestines with or without dextran sulfate sodium (DSS, 3.5%) exposure, fibroblasts (Fb) with or without IL-1 (20 ng/ml) stimulation, bone marrow-derived macrophages (BMDM) with LPS (100 ng/ml) stimulation, and dendritic cells (DC) with or without Pam_3_CSK_4_ (1 μg/mL) or CD40L (1 μg/mL) stimulation. Data are representative of two independent experiments.

High expression of genes in specific cell lines often hints at their importance in these cells and the primary cells from which they derived. We examined relative expression levels of the four mammalian Msrs in various cell lines by examining their gene expression profiles in the BioGPS database (see Materials and Methods) (Fig. 1). Whereas *MSRA*, *MSRB1*, and *MSRB2* were ubiquitously expressed in all cell lines in the dataset, dramatic increases in *MSRB1* expression were observed in some immune cell types. In particular, *MSRB1* expression in BMDMs was potently induced over the course of the LPS response, whereas the expression of other Msr forms was decreased or remained unchanged upon LPS treatment. *MSRB1* was also highly expressed in the mouse macrophage-like cell line RAW264.7. Therefore, these analyses implicated *MSRB1* in the response of macrophages to LPS treatment.

To verify the *MSRB1* expression pattern observed in the BioGPS dataset, we performed quantitative PCR (qRT-PCR) in various cell types following exposure to environmental stress or inflammation-inducing stimuli. This analysis examined *MSRB1* expression in ultraviolet B-irradiated newborn mouse keratinocytes, intestinal epithelial cells isolated from dextran sulfate sodium-administered mice, IL-1-treated mouse embryonic fibroblast (Fb), LPS-treated mouse BMDMs, and Pam_3_CSK_4_/CD40L-treated mouse lymph node dendritic cells (Fig. 1E). An increase in MsrB1 expression was evident only in BMDMs stimulated with LPS.

### MsrB1 is dispensable for LPS-induced intracellular signaling in macrophages

**Figure 2:**
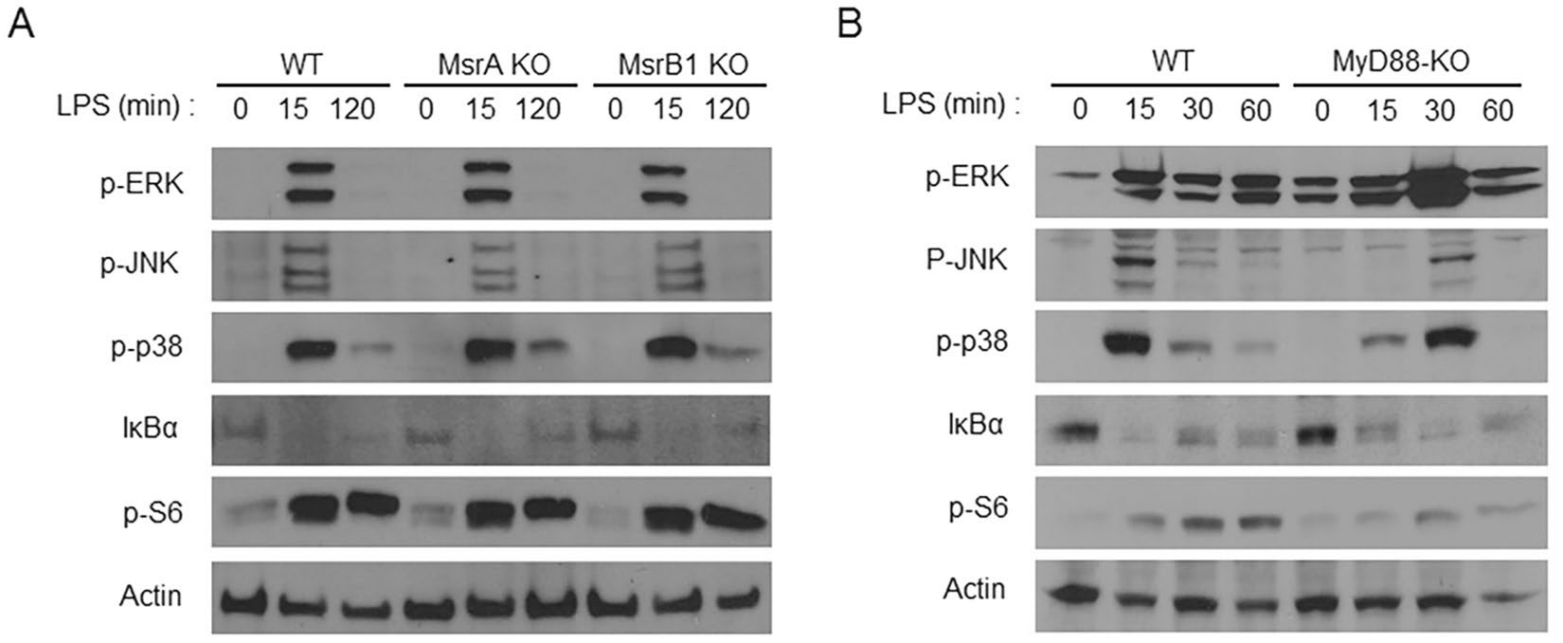
LPS-induced intracellular signaling pathways. (**A**,**B**) Western blot analysis of ERK, JNK, p38, and S6 phosphorylation and IκBα degradation in WT, MsrA KO, and MsrB1 KO BMDMs (**A**) and WT and MyD88 KO BMDMs (**B**) after the indicated duration of LPS stimulation. Cropped gel images are shown and full-length gel images are included in Supplementary Figures S1 and S2.

The high expression of MsrB1 in LPS-stimulated BMDMs may indicate a requirement for its function in macrophages, particularly in relation to their ability to sense and respond to LPS. To address this possibility, we compared LPS-induced intracellular signaling in wild-type (WT) BMDMs and BMDMs derived from mice deficient in particular Msrs. In addition, we compared LPS-induced intracellular signaling in BMDMs from WT and MyD88 KO mice, as deficiency of MyD88, a key intracellular adaptor of the LPS receptor Toll-like receptor (TLR) 4, would represent a major signaling defect in LPS-stimulated cells. To this end, we examined the phosphorylation of ERK, JNK, p38, and S6, and IκBα degradation, all of which represent signaling events downstream of TLR4 (Fig. 2). We found no significant differences in the signaling pathways among WT, MsrA KO, and MsrB1 KO cells, while MyD88 KO cells exhibited a loss of immediate-early induction (15 min after stimulation) of all signaling events examined; in the absence of MyD88, LPS-induced signaling emerged with delayed kinetics. Therefore, neither Msr form was essential for macrophages to detect and trigger intracellular signaling in response to LPS.

### Role of MsrB1 in anti-inflammatory cytokine expression in macrophages

**Figure 3:**
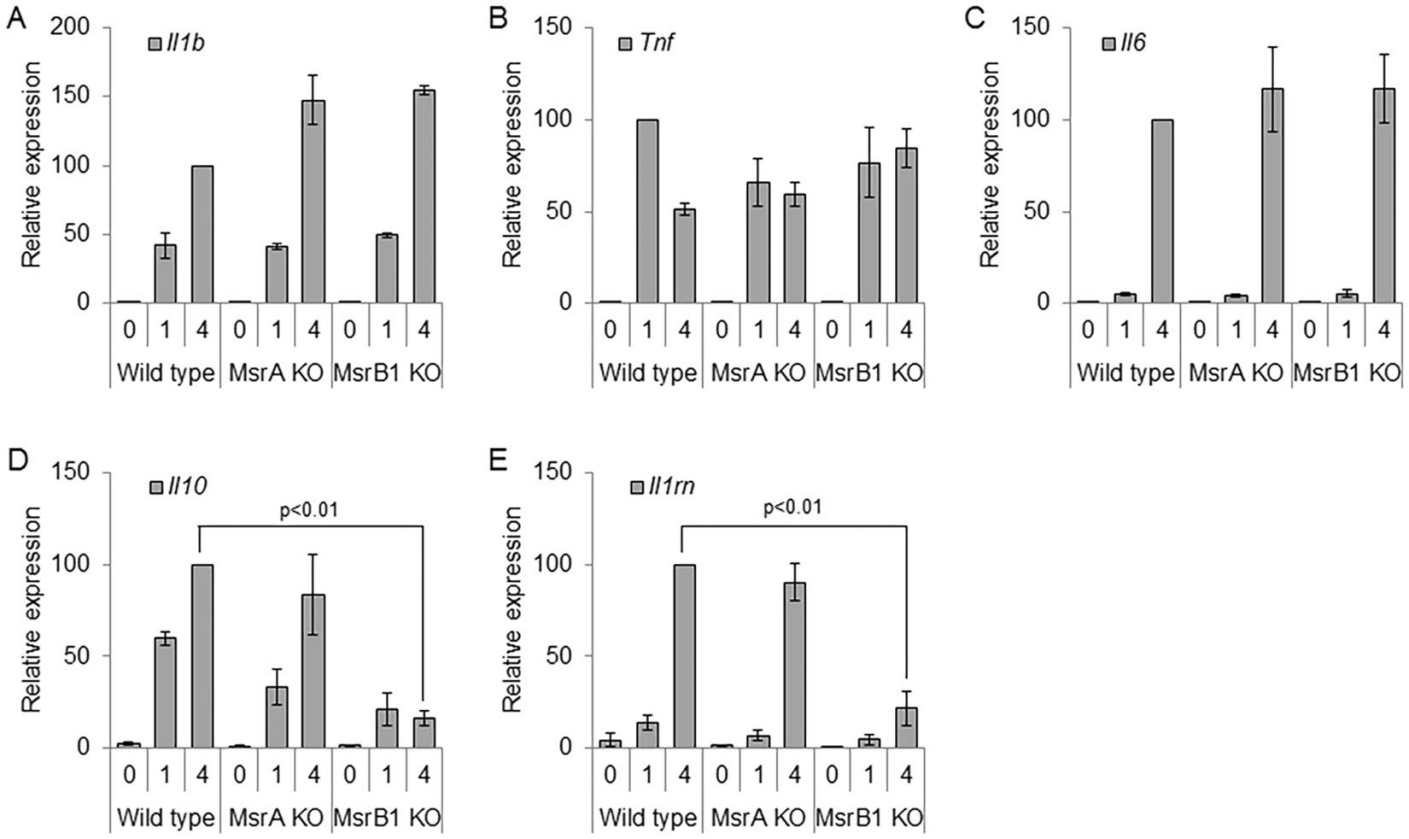
Analysis of cytokine mRNA expression. (**A**–**E**) Relative mRNA amounts for *Il1b* (**A**), *Tnf* (**B**), *Il6* (**C**), *Il10* (**D**), and *Il1rn* (**E**) in WT, MsrA KO, and MsrB1 KO BMDMs were measured after the indicated durations of LPS stimulation. Data are presented as percent values relative to the mRNA amounts at four hours after LPS treatment in WT BMDMs. Error bars represent standard deviation. The experiments were independently repeated two times.

We next examined the effects of Msr deficiency on LPS-inducible gene expression in macrophages. TLR stimulation leads to the activation of complex cascades of intracellular signaling that culminates in a drastic shift in the gene expression program. Among the genes induced by LPS are those encoding pro- and anti-inflammatory cytokines, whose production during the inflammatory response should be kept in balance. We analyzed the expression of a select set of pro- and anti-inflammatory cytokine genes in BMDMs from WT and Msr KO mice (Fig. 3). LPS-induced expression of the pro-inflammatory cytokine genes *Il1b, Tnf, and Il6* in WT and Msr KO cells were largely similar. On the other hand, the induction of the anti-inflammatory cytokine genes *Il10* and *Il1rn* were greatly reduced in LPS-treated MsrB1 KO BMDMs when compared with WT counterparts. By contrast, LPS induction of these two anti-inflammatory cytokine genes was intact in MsrA KO BMDMs.

**Figure 4:**
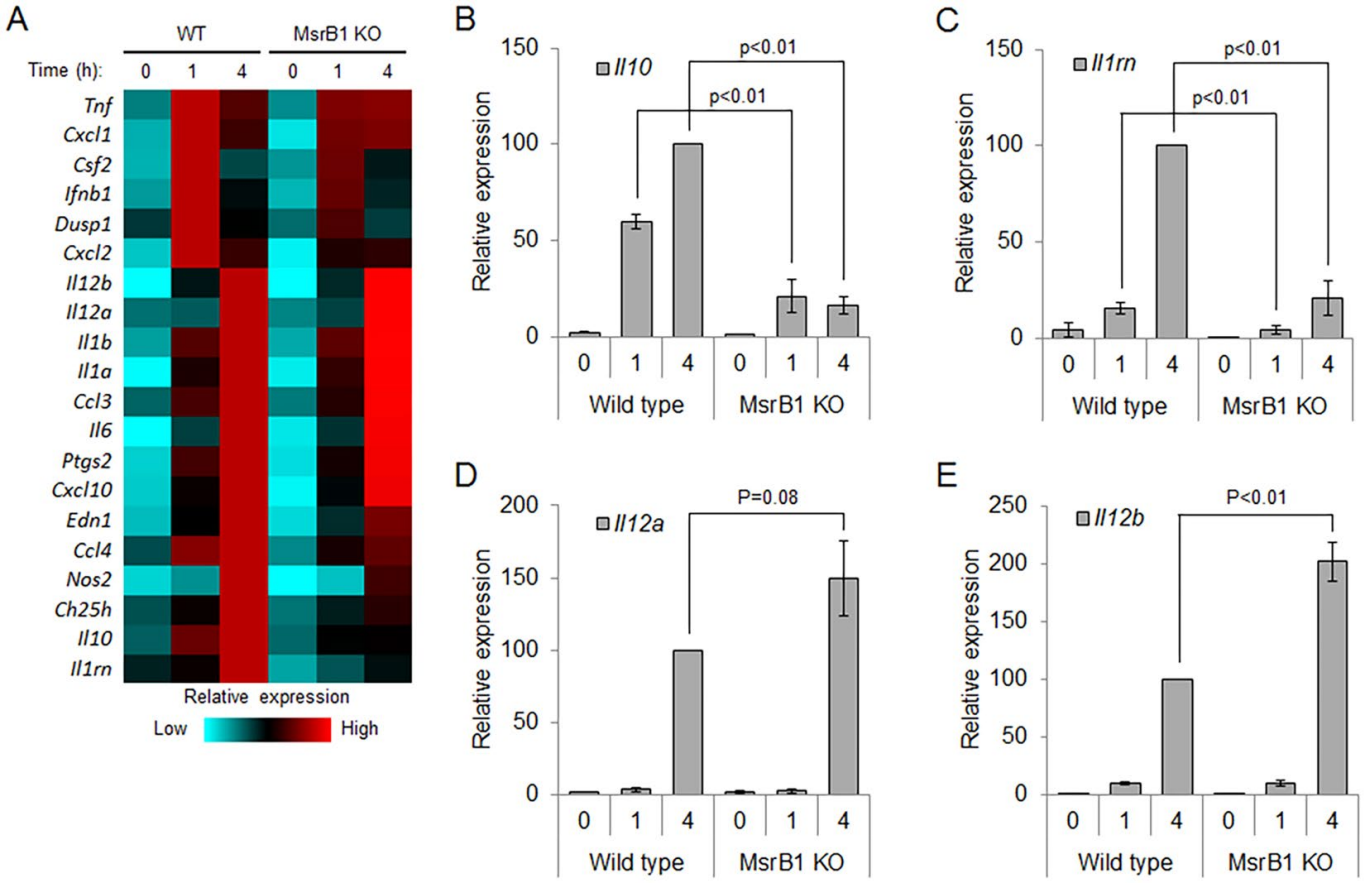
Gene expression profiles in WT and MsrB1 KO BMDMs. (**A**) Relative amounts of mRNA encoding LPS-inducible cytokines, chemokines, and other inflammatory mediators in WT and MsrB1 KO BMDMs were measured after the indicated durations of LPS stimulation. *Tnf* (TNF), *Cxcl1* (CXCL1, GRO1), *Csf2* (GM-CSF), *Ifnb1* (IFN-β), *Dusp1* (DUSP1), *Cxcl2* (CXCL2, GRO2, MIP-2α), *Il12b* (IL-12B, IL-12p40), *Il12a* (IL-12A, IL-12p35), *Il1b* (IL-1β), *Il1a* (IL-1α), *Ccl3* (CCL3, MIP-1α), *Il6* (IL-6), *Ptgs2* (PTGS2, COX-2), *Cxcl10* (CXCL10, IP-10), *Edn1* (ET-1, PPET1), *Ccl4* (CCL4), *Nos2* (NOS2, iNOS), *Ch25h* (h25OH), *Il10* (IL-10), *Il1rn* (IL-1RA) were analyzed. (**B**–**E**) Relative mRNA amounts for *Il10* (**B**), *Il1rn* (**C**), *Il12a* (**D**), and *Il12b* (**E**) were measured as in (**A**). Data is presented as percent values relative to the mRNA amounts at four hours after LPS treatment in WT BMDMs. Error bars represent standard deviation. The experiments were independently repeated three times. Data were statistically analyzed using Student’s t-test (*p* < 0.01).

To determine the effects of MsrB1 deficiency on the expression of a broader set of LPS-inducible genes, we performed qRT-PCR analysis of genes encoding additional cytokines as well as chemokines and other inflammatory enzymes. Intriguingly, this analysis not only confirmed the role of MsrB1 in *Il10* and *Il1ra* induction but also revealed its contribution to attenuating the expression of other LPS-inducible genes, most notably *Il12a* and *Il12b*, as their induction was enhanced in the absence of MsrB1 (Fig. 4). In WT BMDMs, the induction of *Il10* and *Il1rn* (peaked 1 h after LPS treatment) was faster than that of *Il12a* and *Il12b* (peaked 4 h after LPS treatment). In MsrB1 KO BMDMs, reduced expression of *Il10* and *Il1rn* was associated with later enhancement of *Il12a* and *Il12b* expression. A few additional LPS-inducible genes, all pro-inflammatory in their function, exhibited similar increases in expression at 4 h after LPS treatment. Overall, this result supports a potential role of MsrB1 in shaping the LPS-induced gene expression program in macrophages, promoting anti-inflammatory gene expression and limiting the magnitude of pro-inflammatory gene induction.

### Role of MsrB1 in regulating inflammatory responses *in vivo*

**Figure 5:**
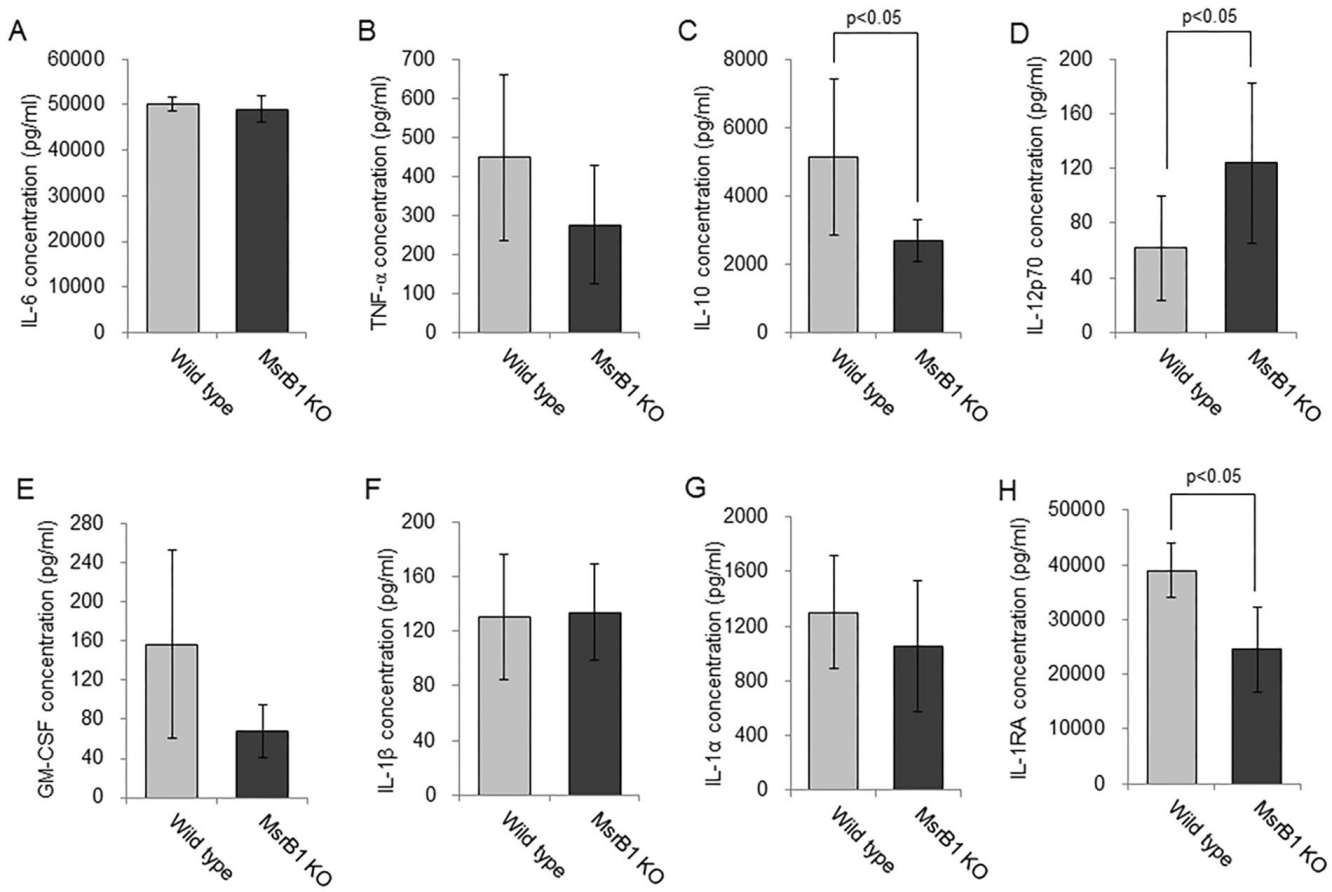
Cytokine protein profiles in WT and MsrB1-KO mouse plasma. (**A**–**H**) Protein concentrations of IL-6 (**A**), TNF (**B**), IL-10 (**C**), IL-12p70 (**D**), GM-CSF (**E**), IL-1β (**F**), IL-1α (**G**), and IL-1RA (**H**) in mouse plasma (n = 8) were measured four hours after intraperitoneal injection of LPS. Error bars represent standard deviation. Data were statistically analyzed by Student’s t-test (*p* < 0.05).

Tissue-resident macrophages and circulating monocytes are key cellular components mediating inflammatory responses *in vivo*. Accordingly, we hypothesized that the reduced expression of *Il10* and *Il1rn* in MsrB1 KO macrophages might affect systemic LPS response *in vivo*. *Il10* and *Il1rn*, encoding IL-10 and the IL-1 receptor antagonist (IL-1RA), play a key role in restraining immune responses in a variety of physiological contexts. In order to explore the *in vivo* effects of MsrB1 deficiency in LPS-administered mice, we measured the concentrations of eight different cytokine proteins (IL-6, TNF, IL-10, IL-12p70, GM-CSF, IL-1β, IL-1α, and IL-1RA) in WT and MsrB1 KO mouse plasma four hours after intraperitoneal injection of LPS (Fig. 5). Consistent with the data on mRNA levels in BMDMs in cell culture, IL-10 and IL-1RA production was reduced in LPS-administered MsrB1 KO mice compared to WT counterparts. Simultaneously, greater amounts of IL-12p70, a cytokine complex composed of the *Il12a* and *Il12b* products were detected in MsrB1 KO plasma. The plasma concentrations of IL-6, TNF, GM-CSF, and IL-1β were comparable in WT and MsrB1 KO mice. These observations further substantiate the role of MsrB1 in inducing IL-10 and IL-1RA in response to LPS. Furthermore, IL-12p70 was overproduced in the absence of MsrB1 both *in vitro* and *in vivo*.

**Figure 6:**
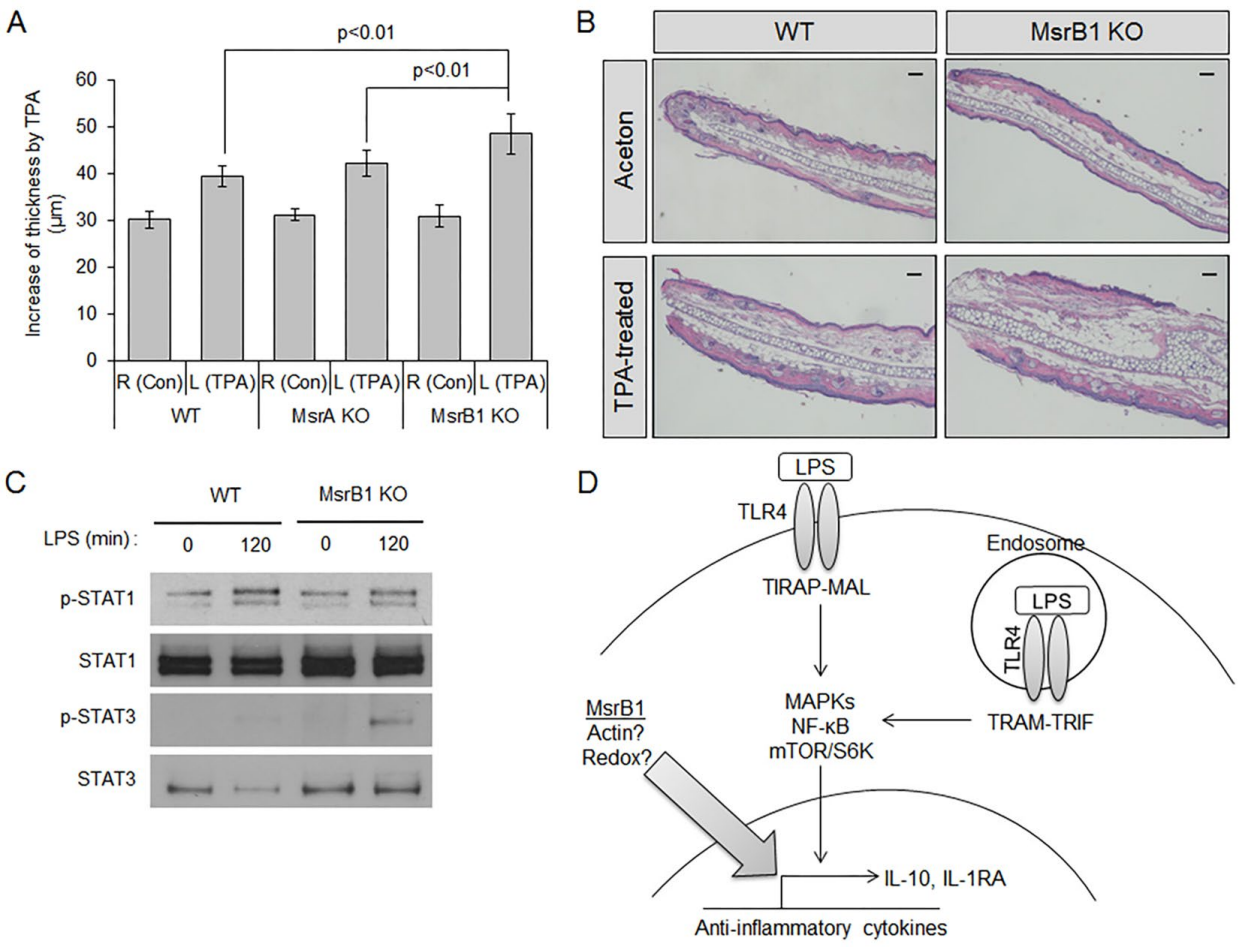
Increased inflammatory responses in TPA-irritated skin of MsrB1-KO mice. (**A**) Skin swelling in TPA-treated left auricles (L) and acetone-treated right auricles (R) was quantified twenty-four hours after irritation (n = 10). Error bars represent standard deviation. Data were statistically analyzed by Student’s t-test (*p* < 0.01). (**B**) Histological features of acetone- and TPA-treated aurticular skin from WT and MsrB1 KO mice were visualized by H&E staining and microscopy (Scale bar = 100 µm). Images are representative of three mice for each treatment group. (**C**) Western blot analysis of STAT1 and STAT3 phosphorylation in WT and MsrB1 KO BMDMs after the indicated duration of LPS stimulation. Cropped gel images are shown and full images are included in Supplementary Figure S3. (**D**) Scheme of MsrB1 function in TLR4 signaling and TLR4-induced IL-10 and IL-1RA expression.

IL-10 and IL-1RA are known to play a key role in suppressing tissue inflammation. Their anti-inflammatory effects in skin inflammation are well established^27–29^. Therefore, the decrease in the expression of these two cytokines in MsrB1 KO mice might cause defects in regulating inflammation in chemically irritated skin. We subjected WT and MsrB1 KO mice to an acute dermatitis model that involved topical treatment of the left auricle with the chemical irritant TPA and the right auricle with acetone (vehicle). Topical TPA, but not acetone, induced tissue edema in all tested animals, resulting in increased thickness in auricular skin (Fig. 6). WT and MsrA KO mice displayed similar extent of skin swelling after TPA treatment without showing statistically significant differences. Meanwhile, MsrB1 KO mice presented with greater severity of TPA-induced skin inflammation with significantly increased skin thicknesses (Fig. 6A). Consistent with this observation, TPA-treated skin of MsrB1 KO mice displayed substantially more enlarged dermal areas than that of WT mice (Fig. 6B). Taken together, the *in vivo* response of MsrB1 KO mice to LPS and TPA revealed that MsrB1 promotes anti-inflammatory cytokine production and limits inflammatory responses *in vivo*.

## Discussion

Apart from the functional effect of MsrB1-mediated redox regulation of actin cytoskeleton dynamics, little is known about the physiological role of this enzyme in macrophages. In this study, we show that MsrB1 is required for the maximal induction of two anti-inflammatory cytokines, IL-10 and IL-1RA, in LPS-stimulated macrophages. Consistent with this *in vitro* finding, MsrB1 was found to contribute to IL-10 and IL-1RA production in mice that received intraperitoneal LPS. Of note, IL-10 is known to enhance LPS-induced IL-1RA expression in macrophages via STAT3 activation^30^. Interestingly, we observed that phosphorylation of STAT3 was enhanced in MsrB1 KO BMDMs, thereby raising the possibility that the functional effect of MsrB1 on the induction of the two anti-inflammatory cytokines is sequential rather than parallel (Fig. 6C). The role of MsrB1 in anti-inflammatory cytokine expression was associated with enhanced expression of pro-inflammatory mediators, most notably IL-12. These alterations in cytokine production likely account for enhanced tissue inflammation in MsrB1 KO mice. All of these *in vitro* and *in vivo* changes were not visible in mice with MsrA deficiency, indicating that the immune regulatory properties of MsrB1 are distinct from those that MsrA may have in macrophages.

In all likelihood, MsrB1 promotes anti-inflammatory cytokine production at the level of transcription or a post-transcriptional process affecting mRNA abundance. It remains to be determined precisely how MsrB1 exerts this function. TLR4 stimulation by LPS leads to the induction of pro- and anti-inflammatory cytokines in a manner dependent on the intracellular adaptor complexes TIRAP-MyD88 and TRAM-TRIF. These adaptor complexes in turn transmit activation signals to multiple protein kinase and transcription factor modules such as those involving NF-κB, ERK, JNK, p38, and mTOR/S6K. Given that signaling via all of these modules was intact in MsrB1 KO macrophages, MsrB1 is likely to exert its functional effects on IL-10 and IL-1RA expression via as-yet-unidentified signaling mechanisms (Fig. 6D). The function of MsrB1 to control actin assembly could be related to the MsrB1-dependent mechanism promoting IL-10 and IL-1RA induction. Indeed, there is evidence showing a link between actin cytoskeleton dynamics and immune response gene expression in macrophages^31, 32^. As an alternative mechanism, MsrB1 may play a role in the redox modification of other, non-actin substrate proteins that are crucial for cytokine gene expression.

We have identified MsrB1 as a novel regulator of macrophage immune function. This discovery reveals a new layer of molecular control in immune signaling. It is expected that MsrB1 function is based on its reductase activity, which makes it an actionable target for modulation by small-molecule compounds. Moreover, MsrB1 is a selenoprotein that is readily regulated by dietary selenium. Although in principle we cannot rule out a non-enzymatic function of MsrB1, pharmacological or dietary inhibition of the reductase activity of MsrB1 or the oxidase activity of its antagonistic partner, Mical, is a therapeutic approach worth pursuing.

## Electronic supplementary material

Supplementary Information
