# Supplementary material for: Selenoprotein MsrB1 promotes anti-inflammatory cytokine gene expression in macrophages and controls immune response in vivo
Source: Sci Rep. 2017 Jul 11;7:5119. doi: 10.1038/s41598-017-05230-2 (PMC5506048; doi:10.1038/s41598-017-05230-2)
Supplement: Supplementary file 1 — Supplementary Information [file 41598_2017_5230_MOESM1_ESM.pdf]

Selenoprotein MsrB1 promotes anti-inflammatory cytokine gene expression in macrophages and controls immune response *in vivo*

Byung Cheon Lee[1], Sang-Goo Lee[2], Min-Kyung Choo[3], Ji Hyung Kim[4], Hae Min Lee[1], Sorah Kim[1], Dmitri E. Fomenko[5], Hwa-Young Kim[6], Jin Mo Park[3], and Vadim N. Gladyshev[2]

[1]College of Life Sciences and Biotechnology, Korea University, Seoul, 02841, South Korea; [2]Division of Genetics, Department of Medicine, Brigham & Women's Hospital and Harvard Medical School, Boston, MA 02115, USA; [3]Cutaneous Biology Research Center, Massachusetts General Hospital and Harvard Medical School, Charlestown, MA 02129, USA; [4]Program in Cellular and Molecular Medicine, Boston Children's Hospital, Department of Microbiology and Immunobiology, Harvard Medical School, Boston, MA 02115 USA; [5]Department of Biochemistry and Redox Biology Center, University of Nebraska, Lincoln, NE 68588, USA; [6]Department of Biochemistry and Molecular Biology, Yeungnam University College of Medicine, Daegu, 42415, South Korea

Table S1. Oligonucleotide primers used in qPCR.

Gene	Forward primer (5' to 3')	Reverse primer (5' to 3')
Ccl3	ccaagtcttctcagcgccat	tccggctgtaggagaagcag
Ccl4	gacttggagttgaactgagcagc	aggcctctcctgaagtggc
Ch25h	ccacgacatgcatcactctc	gcattttgtcccagtgtgtg
Csf2	ggccttggaagcatgtagag	gcatgtcatccaggaggttc
Cxcl1	gccaatgagctgcgctgt	ccttcaagctctggatgttcttg
Cxcl2	atccagagcttgagtgtgacgc	aaggcaaactttttgaccgcc
Cxcl10	gaatccggaatctaagaccatcaa	gtgcgtggcttcactccagt
Dusp1	ccatctgccttgcttacctc	aagctgaagttcggggagat
Edn1	aaggcatcttttcgtgttgc	ttgtgcgtcaacttctggtc
Ifnb1	cgtgggagatgtcctcaact	acctttgcaccctccagtaa
Il1a	tccagggcagagagggagt	ggaactttggccatcttgattt
Il1b	gtggctgtggagaagctgtg	gaaggtccacgggaaagacac
Il1rn	ttacaaggaccaaatatcaaactagaag	ggatgcccaagaacacactatg
Il6	ccagaaaccgctatgaagttcc	ttgtcaccagcatcagtccc
Il10	gaagctgaagaccctcagga	ttttcacaggggagaaatcg
Il12a	cagaaacctcctgtgggaga	ggagctcagatagcccatca
Il12b	atccagcgcaagaaagaaaa	ggaacgcacctttctggtta
Msrb1	agcgttcactgaaaccatcc	ttttctcagggcacttggtc
Nos2	caagcaccttggaagaggag	ccaaatgtgcttgtcaccac
Ppia	atggtcaaccccaccgtgt	ttcttgctgtctttggaactttgtc
Ptgs2	cccccacagtcaaagacact	ggttctcagggatgtgagga
Tnf	acagaaagcatgatccgcg	gccccccatcttttggg

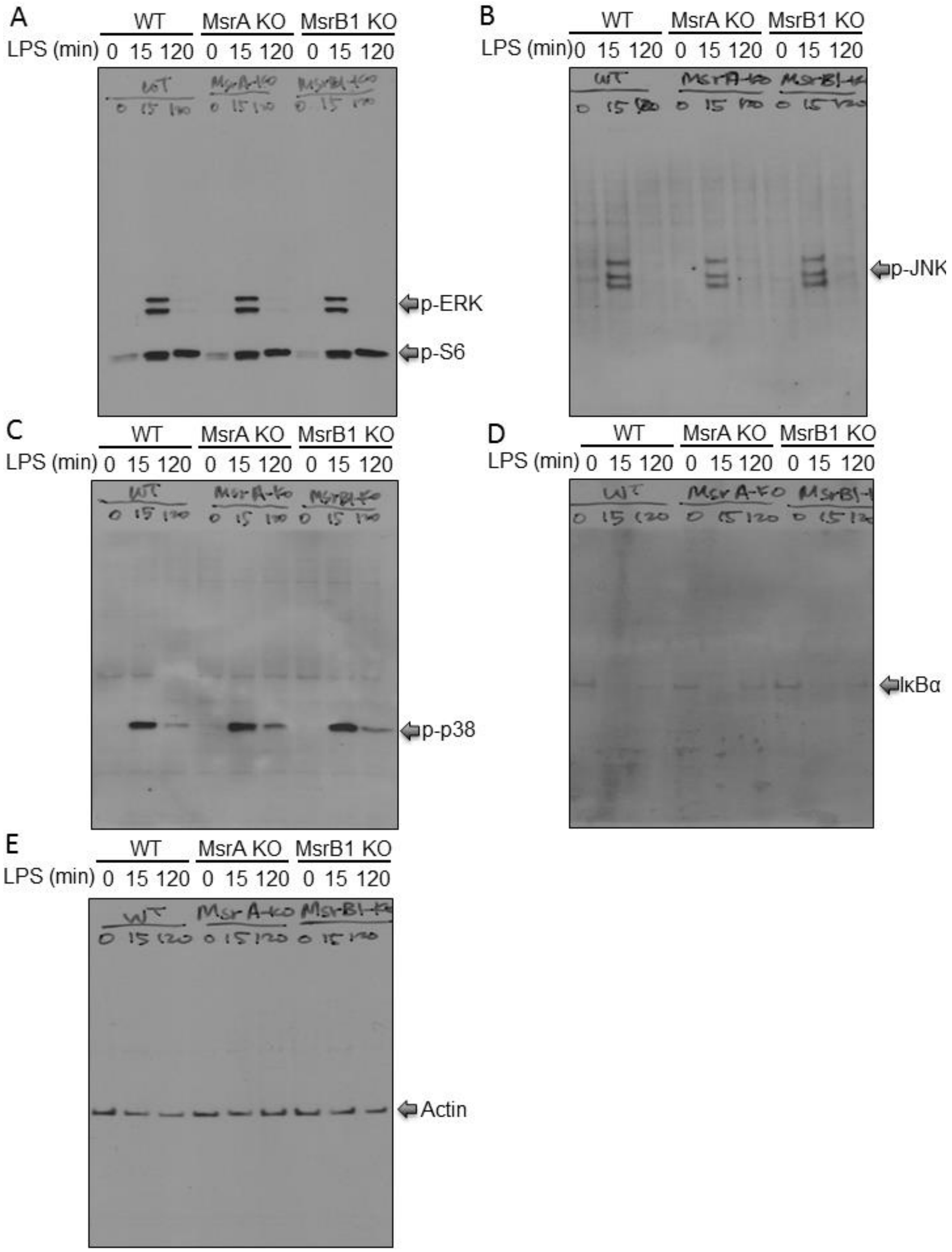

Supplementary Figure S1. Full-length image of western blot analysis. (A-E) All figures are full-length image of western blot analysis of ERK, JNK, p38, and S6 phosphorylation and IκBα degradation in WT, MsrA KO, and MsrB1 KO BMDMs after the indicated duration of LPS stimulation in Figure 2.

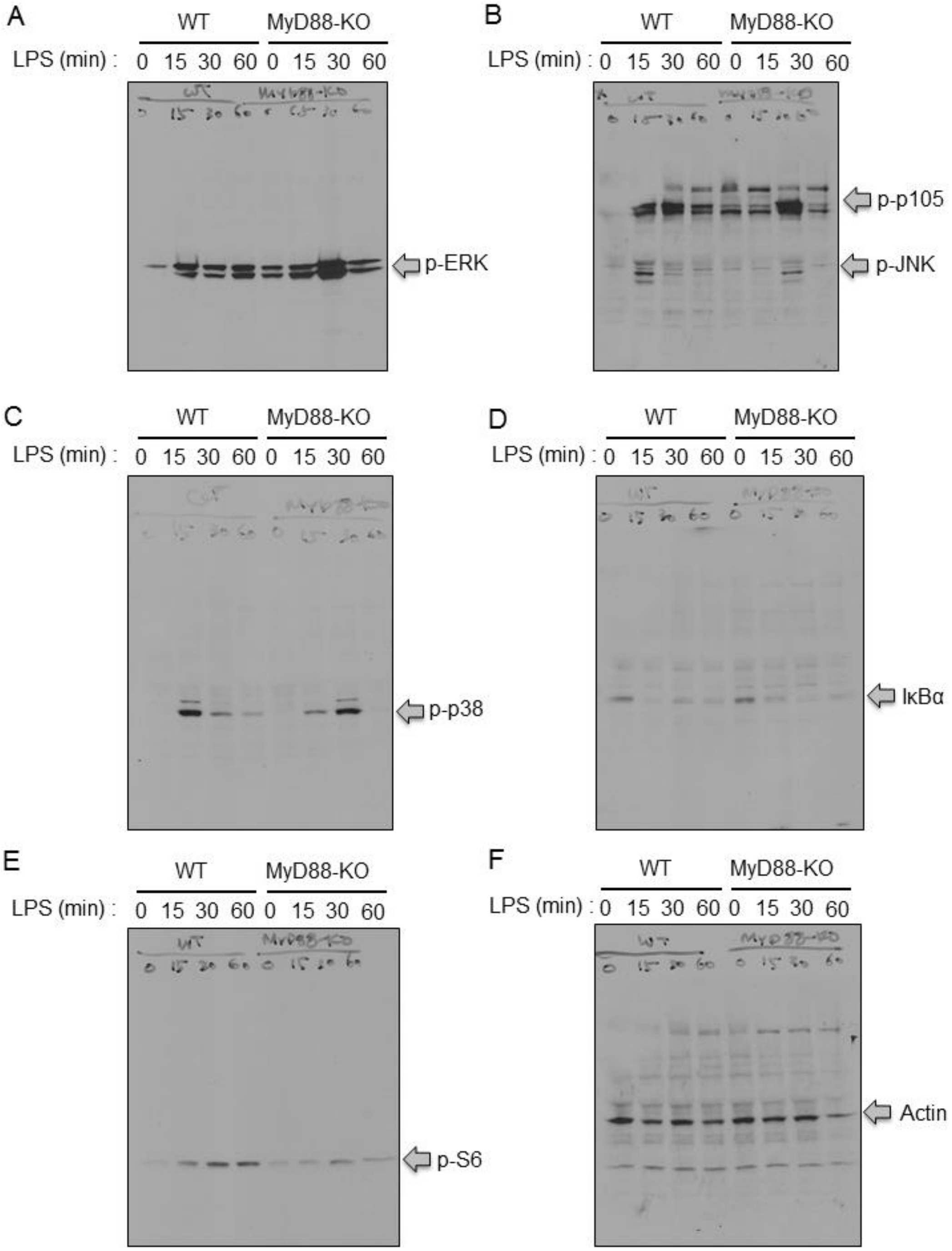

Supplementary Figure S2. Full-length image of western blot analysis. (A-F) All figures are full-lenth image of western blot analysis of ERK, JNK, p38, and S6 phosphorylation and IκBα degradation in WT and MyD88 KO BMDMs after the indicated duration of LPS stimulation in Figure 2.

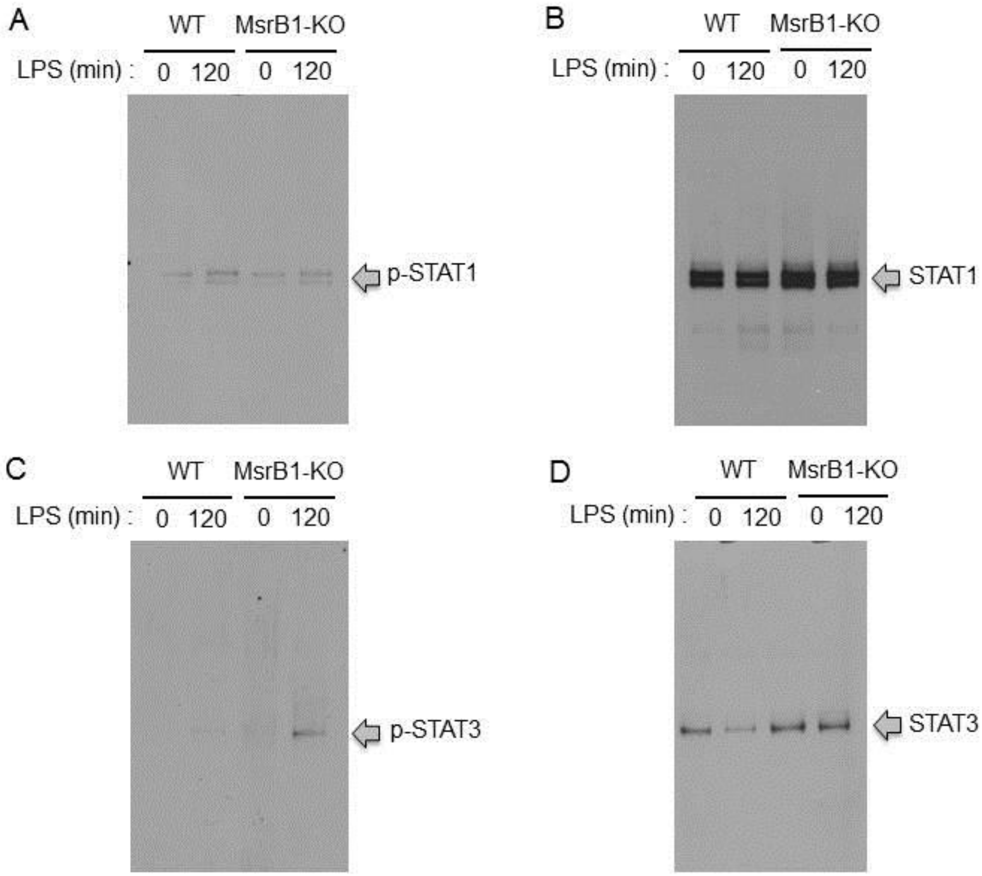

Supplementary Figure S3. Full-length image of western blot analysis. (A-D) All panels are full images representing western blot analysis of STAT1 and STAT3 phosphorylation in WT and MsrB1 KO BMDMs after the indicated duration of LPS stimulation (Figure 6C).
